# Early EEG responses to pre-electoral survey items reflect political attitudes and predict voting behavior

**DOI:** 10.1038/s41598-021-96193-y

**Published:** 2021-09-21

**Authors:** Giulia Galli, Davide Angelucci, Stefan Bode, Chiara De Giorgi, Lorenzo De Sio, Aldo Paparo, Giorgio Di Lorenzo, Viviana Betti

**Affiliations:** 1 Department of Psychology, Kingston University, Kingston, UK; 2 Department of Political Science, LUISS Guido Carli, Rome, Italy; 3 Melbourne School of Psychological Sciences, The University of Melbourne, Melbourne, Australia; 4 IRCCS Fondazione Santa Lucia, Rome, Italy; 5 Department of Psychology, “Sapienza” University of Rome, Rome, Italy; 6 Laboratory of Psychophysiology and Cognitive Neuroscience, Department of Systems Medicine, University of Rome “Tor Vergata”, Rome, Italy

**Keywords:** Decision, Social behaviour, Social neuroscience

## Abstract

Self-reports are conventionally used to measure political preferences, yet individuals may be unable or unwilling to report their political attitudes. Here, in 69 participants we compared implicit and explicit methods of political attitude assessment and focused our investigation on populist attitudes. Ahead of the 2019 European Parliament election, we recorded electroencephalography (EEG) from future voters while they completed a survey that measured levels of agreement on different political issues. An Implicit Association Test (IAT) was administered at the end of the recording session. Neural signals differed as a function of future vote for a populist or mainstream party and of whether survey items expressed populist or non-populist views. The combination of EEG responses and self-reported preferences predicted electoral choice better than traditional socio-demographic and ideological variables, while IAT scores were not a significant predictor. These findings suggest that measurements of brain activity can refine the assessment of socio-political attitudes, even when those attitudes are not based on traditional ideological divides.

## Introduction

The standard approach to the measurement of political preferences ahead of an election consists of using surveys in which respondents are explicitly asked to express their views. However, there are several limits to the use of self-reports^1,2^. Respondents may be unwilling to share their thoughts and feelings or may be unable to verbalize them^3^. As a consequence, responses on self-report measures can be distorted by social desirability and self-presentation bias^4^. Furthermore, self-reports are unable to capture attitudes or preferences that are outside conscious awareness. These limitations can be addressed by methods that measure attitudes indirectly or implicitly. Psychological research has demonstrated that implicit measurements can capture attitudes above and beyond self-reports, especially socially sensitive attitudes^5,6^. Within the realm of political preferences, one of the most widely used tests to measure implicit attitudes is the Implicit Association Test (IAT), a method that indirectly measures the strength of associations among concepts^7^. Across different countries, IAT scores have predicted undecided people’s opinions on politically-charged matters^8^, future voting choices^9^, and has even improved electoral forecast at a national level^10^. The predictive validity of the IAT in electoral contexts, however, is not always superior to explicit measurements of voting intentions, such as simply asking respondents who they will vote for^9^. In addition, the test falls short in providing insights into the complex dynamics of political attitudes and voting decisions.

More recently, there has been a burgeoning interest in the measurement of political attitudes using brain activity^11–13^. Functional Magnetic Resonance Imaging (fMRI) and electroencephalography (EEG) studies have revealed differences in brain function depending on partisanship or political preferences, providing information on the mechanisms underlying those attitudes^14–18^, and predicting actual voting behavior^19^. Non-invasive brain stimulation techniques have also clarified the causal role of brain regions in implicit ideologies or attitudes^20^. Similar to the IAT, these methods measure political preferences and attitudes by bypassing deliberate, and often distorted, responses. Given the rapidly growing interest in the social neuroscience of political attitudes, it is timely to consider how the measurement of political attitudes using brain activity compares to other implicit and explicit methods of attitude measurement.

In this study we compared different predictors of voting choice in a real electoral context. We used EEG as measurement of brain activity for two main reasons. Firstly, theoretical frameworks of social attitudes evaluation suggest that socio-political concepts are rapidly and automatically activated upon the presentation of an attitude-related stimulus^21–25^. This initial processing, involving affective-related limbic structures, is followed by more controlled processing, recruiting higher-order cortical areas^25^. Differences in political attitudes may thus result from implicit and explicit measures capturing the current attitude evaluation at different stages of information processing^12^. A technique with excellent temporal resolution is therefore particularly suitable to compare how the measurement of political attitudes using brain activity compares with other methods of attitude evaluation. Secondly, in our previous investigation we found that the N400 indexed political preferences in the context of the 2016 EU referendum in the UK^19^. The N400 is a negative deflection of the event-related potential (ERP) typically observed 300–600 ms over posterior channels. This component is traditionally considered an index of semantic incongruency^26^, but several social neuroscience studies have demonstrated that this component is also responsive to incongruencies with one’s moral values, stereotypes^27–29^ and violations of social norms^30^. Consistent with this functional interpretation, in our previous study we found that the N400 was larger for information that contradicted participants’ views on the EU, and its modulation predicted future referendum voting behavior^19^. Thus, in the current investigation, we used the N400 as an index of political preferences and as a brain-based predictor of voting behavior.

Importantly, the current study focuses on populist attitudes, which so far have received little attention from psychological and neuroscientific literature, which is largely dominated by liberal-conservative or left–right ideological divides. Yet, in many Western countries the current political debate is increasingly structured on the opposition between populist and mainstream parties, and a surge in populist sentiments is evident in politics as well as in these societies^31^. In general, populist parties are characterized by several key elements, such as people-centrism, anti-elitism, and anti-pluralism—all elements configuring a common anti-establishment denominator. However, populism can cut across the left–right dimension and therefore can be hosted by different ideologies, resulting in both right-wing and left-wing populist parties^32,33^. This characteristic makes populism an interesting dimension to investigate from a neurobiological perspective. The political division described by the labels “liberal” and “conservative”, or “left” and “right”, is ancient and possibly universal^34,35^. It is thus unsurprising that this division is associated with different neurobiological substrates, from brain structure and function^11–13,36^ to genetic heritage^37^. An open question is whether neurobiological differences can also be observed in other and relatively more recent political divides, such as that which opposes populist parties to traditional mainstream parties.

To address these issues, during EEG recording 82 future voters at the European Parliament election in Italy (May 26 2019) expressed their agreement or disagreement with a number of survey items expressing populist and non-populist views. After the recording session, they completed an IAT on attitudes towards the leaders of the main populist and mainstream political parties at the time of the election. We compared the predictive validity of the two implicit indices—EEG responses, IAT scores—with two explicit indices. Explicit indices consisted of a set of socio-demographic predictors that were used in electoral research, and the self-reported agreement with the survey items during the EEG session. We examined political attitudes using both an event-related potential (ERP) component approach and a multivariate pattern analysis (MVPA). We hypothesized that populist views would be instantiated in the brain, and that features associated with populist and non-populist narratives would be rapidly and automatically extracted from the survey items^21–25^*.* Based on our previous investigation^19^, we expected that the disagreement with those views would trigger a modulation of the N400. Specifically, we predicted that the N400 would be larger in response to items that contradicted one’s political attitudes (e.g., “For the future of our country, elitism is beneficial” in a voter of a populist party). MVPA provided an additional unbiased investigation of brain signals using the entire spatio-temporal pattern of ERPs as input, therefore allowing us to decode whether each survey item expressed populist or non-populist narratives from distributed spatio-temporal patterns of brain activity^38^. We expected that brain activity elicited by issues that are more relevant to the narratives of mainstream and populist parties would show higher decoding accuracy and possibly processing priority. Crucially, based on previous evidence in both political and other social domains, we hypothesized that brain responses would predict real-world choice, namely voting for a populist or a mainstream party, above and beyond explicit measures^19,39,40^.

## Results

### Voting behavior

Of the 82 collected participants, 41 later voted for a mainstream party (26 females; M_age_ = 23 years; SD_age_ = 4 years; 21 undecided participants at the time of testing) and 28 later voted for a populist party (11 females; M_age_ = 25 years; SD_age_ = 6 years; 9 undecided participants at the time of testing). Thirteen participants were excluded from the analyses because they voted for a party that could not be classified as mainstream or populist (9 participants), they did not vote (3 participants) or they returned a blank ballot paper (1 participant). The final sample thus consisted of 69 participants (40 voters of a mainstream party, 29 voters of populist party). The assignment of parties to the mainstream or populist group was based on previous work on Italian parties^41,42^.

### Self-report

To analyze self-reported preferences, we analyzed the rate of agreement with the survey items during the experiment and calculated a self-report index (SRI) adapting the Kelley index^43^ used in American pre-electoral surveys:

$$\mathrm{SRI} = \left(\% \; Populist \; items \; agreed+\% \; Mainstream \; items \; disagreed \right)- \left(\% \; Populist \; items \; disagreed+\% \; Mainstream \; items \; agreed\right)$$

**Figure 1:**
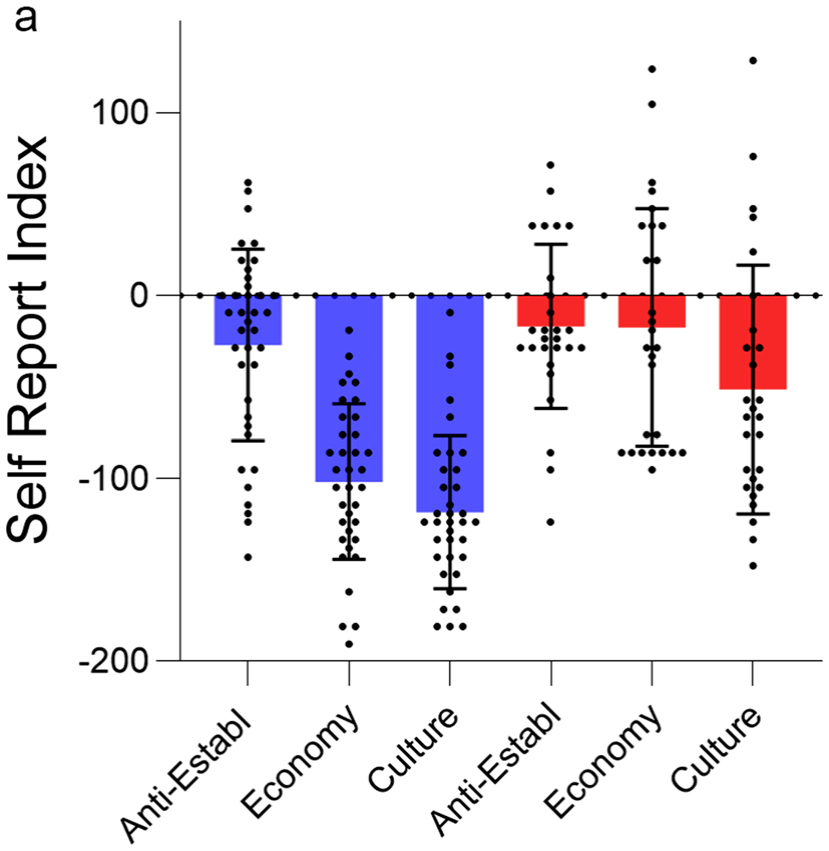
Task performance. SRI for the three issue dimensions in voters of mainstream (blue) and populist (red) parties. More positive values correspond to more populist views. The SRI was polarized for economy and culture issues, but not for anti-establishment ones.

Note that SRI values expressed the extent to which one supports populist or non-populist views, but the 0 mark should not be considered a cutoff score to separate individuals with or without a populist ideology. An ANOVA with Voting Behavior (two levels: Mainstream, Populist) as the between-subjects factor and Issue Dimension (three levels: Anti-Establishment, Economy, Culture) as the within-subjects factor resulted in a significant Voting Behavior × Issue Dimension interaction (F_2,130_ = 12.33, p < 0.001, η_p_^2^ = 0.159; note that data from two participants were not collected due to technical difficulties). As shown in Fig. 1, self-reported preferences on Economy and Culture were more polarized and differed between voters of mainstream and populist parties (Bonferroni-corrected independent-samples t-test, Economy t_65_ = 6.27, p < 0.001, d = 1.55; Culture t_65_ = 5.07, p < 0.001, d = 1.26) whereas for Anti-establishment there was no difference between the groups (p = 0.361). There was also a main effect of voting behavior indicating that, as expected, the SRI was more negative for mainstream voters (F_1,65_ = 31.88, p < 0.001, η_p_^2^ = 0.329). Mean RTs for each condition were computed excluding trials where subjects took more than 2700 ms to respond (2 SDs above the average untrimmed RT across all conditions) and collapsing across agreement and disagreement responses. An ANOVA with Voting Behavior (two levels: Mainstream, Populist) as the between-subjects factor, and Issue Dimension (three levels: Anti-Establishment, Economy, Culture) and Item Type (two levels: Populist, Non-populist) as the within-subjects factors, did not result in any significant main effect or interaction involving Voting Behavior, Issue or Item Type (ps > 0.144), suggesting that any difference in brain responses between the two groups was not related to group differences in general processing speed (Supplementary Table S1).

### N400 effects in voters of mainstream and populist parties

**Figure 2:**
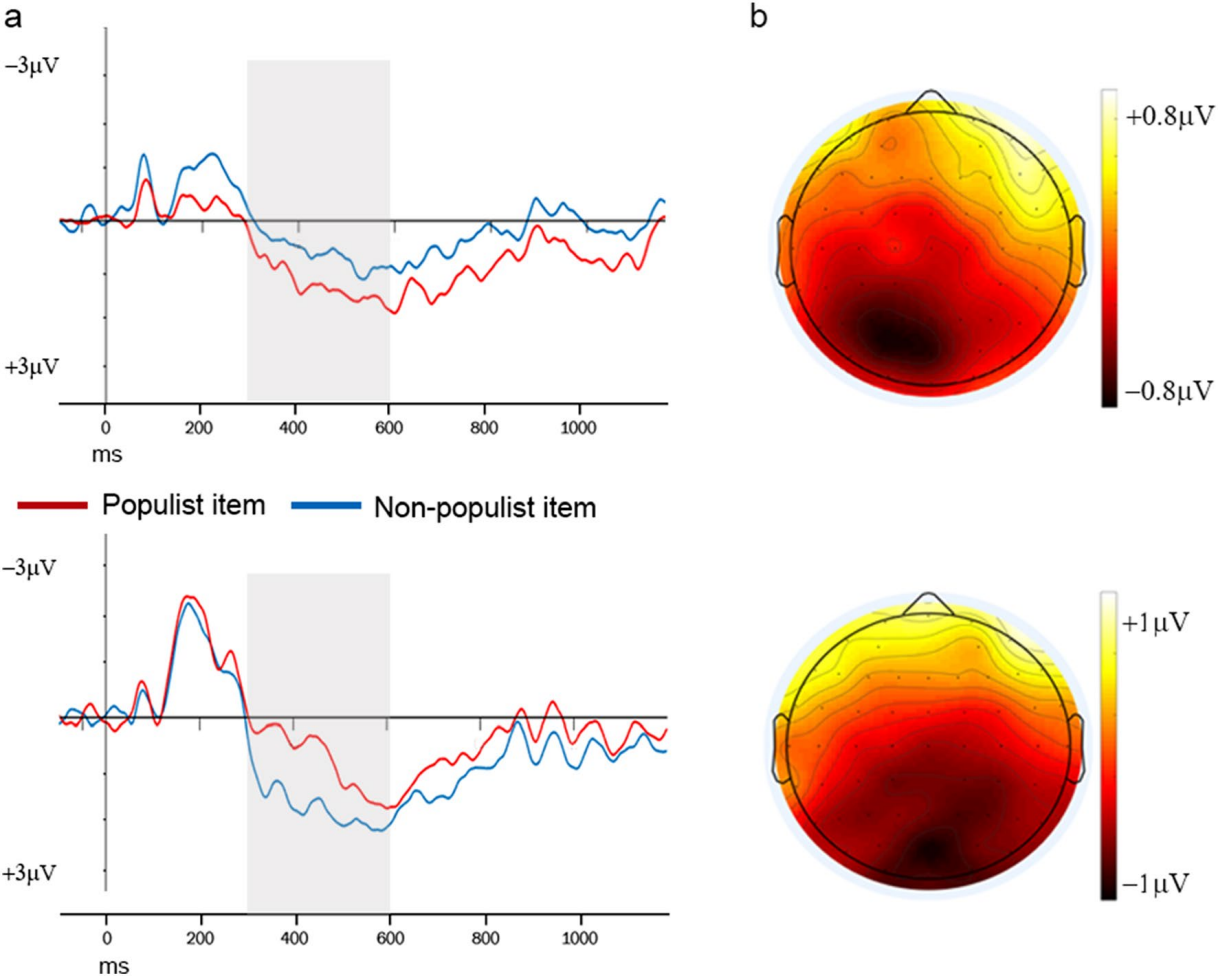
N400 in voters of mainstream and populist parties. (**a**) N400 effects in voters of populist (above) and mainstream (below) parties for economy survey items. All waveforms are from electrode POz, where the effects were most prominent. The grey box illustrates the time window used for statistical analyses. Note that negative is plotted upwards. (**b**) Topographic maps representing the scalp distribution of the observed ERP difference between congruent and incongruent items for economy items in the 300–600 ms latency region in voters of populist (above) and mainstream (below) parties.

Based on the functional significance of the N400 and our previous findings^19,26–30^, we hypothesized that the N400, time-locked to the last word completing a survey item, would be larger for item types that contradicted one’s political attitudes (i.e., larger for survey items that expressed non-populist views in voters of populist parties, and larger for survey items that expressed populist views in voters of mainstream parties). This hypothesis was tested with a mixed model ANOVA with Voting Behavior (two levels: Mainstream, Populist) as the between-subjects factor, and Item Type (two levels: Populist, Non-Populist), Issue Dimension (three levels: Anti-Establishment, Economy, Culture) and Electrode (12 levels) as the within-subjects factors. The ANOVA revealed a Voting Behavior × Item Type × Issue Dimension interaction (F_2,130_ = 6.05, p = 0.003, ε = 0.993, η_p_^2^ = 0.085). To decompose this interaction, we first investigated which issue dimension elicited N400 effects and performed follow-up ANOVAs separately for each issue dimension. The Voting Behavior × Item Type interaction was significant for the Economy (F_1,65_ = 15.18, p < 0.001, η_p_^2^ = 0.189) but not for the Anti-establishment and Culture dimensions (p = 0.726 and p = 0.832, respectively), indicating that N400 effects differed between voters of mainstream and populist parties only when the survey items covered economic issues. Two follow-up ANOVAs, performed separately on the two groups of voters, showed that the N400 for Economy was more negative-going for populist items in mainstream voters, and for non-populist items in populist voters (main effect of Item Type, mainstream voters: F_1,39_ = 11.32, p = 0.002, η_p_^2^ = 0.225; populist voters: F_1,26_ = 5.66, p = 0.025, η_p_^2^ = 0.179), therefore confirming our hypothesis that the N400 is larger for survey items that disagreed with one’s political attitudes (Fig. 2a,b).

### Multivariate pattern analysis (MVPA)

**Figure 3:**
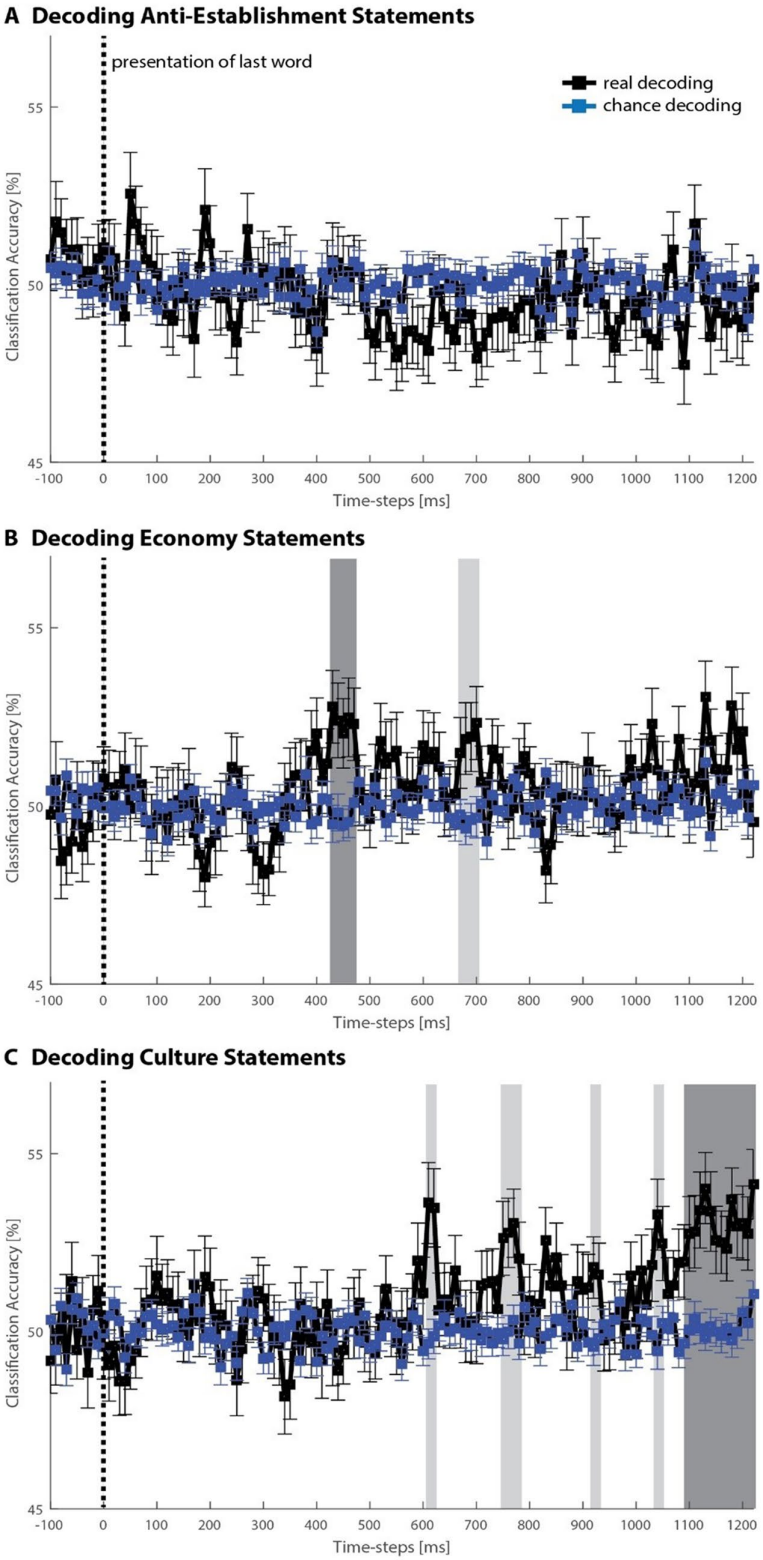
Multivariate pattern classification. EEG data results (N = 67) for the decoding of populist vs. non-populist items for the three political issue dimensions: (**A**) Anti-establishment, (**B**) economy, (**C**) culture. Significant classification indicates that neural patterns in small analysis windows of 10 ms allowed for the prediction of whether an item on a given trial expressed populist or non-populist views. Dark grey bars indicate significant clusters (minimum two time windows) after correction for multiple comparisons; light grey bars indicate significant clusters uncorrected (p < 0.05).

MVPA is highly sensitive to information represented in the overall signal as it uses the entire spati + o-temporal pattern of ERPs across all channels as input^38,44,45^, thereby allowing for the extraction of features from the EEG that are not dependent on the identification of ERP components. We used MVPA to examine whether neural representations could predict whether a given survey item expressed populist or non-populist views for each political issue dimension. We reasoned that items that elicited stronger feelings of agreement or disagreement due to one’s underlying political beliefs would be more strongly represented in neural patterns and, as a consequence, increase the accuracy for predicting the item type (that is, populist and non-populist survey items) from brain activity. For this analysis, the data was first separated by political issue category (anti-establishment, economy, and culture). For each of these categories, all single-trial data was then sorted by item type (populist vs. non-populist survey items), and a series of separate support vector machine (SVM) classification analyses was conducted on data from small 10 ms analysis time windows, which were moved through the trial. Each time, the classifier was trained on 90% of all data within the analysis time window, from both populist and non-populist items. The remaining, independent 10% of the data were used to predict the item type. The final prediction accuracy (i.e. how well the classifier generally predicted populist from non-populist items across trials) for each political issue category was calculated by averaging across ten independent iterations of a tenfold cross-validation process, in which each data set was used for testing once while training on all other data. Group-level statistical testing was performed against an empirical shuffled-labels (i.e. chance) distribution for each analysis window separately. For this, we used t-tests (α = 0.05) for each analysis time window, and corrected for multiple-comparisons (i.e. the number of analysis time windows) using cluster-based permutation tests based on the cluster mass statistic (cluster inclusion alpha = 0.05, permutation samples = 5000; see “Materials and methods” for details). Figure 3 shows the predictive accuracy for the three political issue dimensions. Significant prediction was found at 430–470 ms for cluster-corrected economy survey items, starting at 1080 ms until the end of the analyzed trial period for culture survey items. Other significant clusters emerged, however these did not survive Bonferroni correction (Supplementary Table S2). No significant prediction from anti-establishment items emerged. These results did not differ when we used 20 ms analysis time windows instead of 10 ms as a sanity check. Altogether, MVPA showed that the political content of the item in terms of populist or non-populist narrative could be decoded from brain activity at an early stage of processing for economy items, and at a later stage of processing for culture items.

### IAT

To evaluate IAT performance, we calculated the D index following the procedures outlined in Greenwald, Nosek and Baniji^46^. First, we deleted trials greater than 10,000. We then computed the inclusive standard deviation of the latencies separately for combined blocks 3 and 6 and for blocks 4 and 7 (see Supplementary Table 4) and the average latency for each one of those blocks. Furthermore, we computed the difference between the average latencies (Mean_block6_ − Mean_block3_) and (Mean_block7_ − Mean_block4_) and divided each difference score with its corresponding inclusive standard deviation. D was the equal-weight average of the two resulting ratios. All subjects had latencies above 300 ms. More positive scores corresponded to more positive attitudes towards populist leaders. To assess internal consistency, we calculated the correlation between the D index for the first combined blocks and the D index for the second combined blocks. The Spearman-Brown adjusted split-half correlation was r = 0.76, suggesting a satisfactory reliability. There was no difference in attitudes between voters of populist (M = 0.25, SD = 0.36) and mainstream (M = 0.22, SD = 0.55) parties (p = 0.875).

### Comparison of predictors

Finally, we compared different predictors of voting behavior. Implicit predictors were the N400 effect for Economy and scores at the IAT. For the N400, we used the averaged differential waveform between populist and non-populist items for Economy at POz, where the effect was largest. Explicit predictors were the SRI for Economy and a classic voting model, including socio-demographic and traditional political and ideological predictors (age, gender, left–right self-placement and interest in politics), recorded with a political orientation questionnaire which was administered at the beginning of the experiment (see “Materials and methods”). We also tested a model incorporating both the N400 and the SRI. A logistic regression was utilized due to the dichotomous nature of the dependent variable (voters of mainstream vs populist parties). The comparison of different models is shown in Table 1. IAT scores (Model 2) were not predictive of voting choice. Instead, the N400 (Model 1) showed a statistically significant effect on electoral behavior (λ—adjusted correctly predicted count) which was independent from the effect of the SRI (Model 3). Overall, when considering the N400 and the SRI in isolation, the SRI appeared more relevant than the N400 in predicting the vote, as the magnitude of the coefficients and the goodness-of-fit measures suggests. However, a Wald test of the equality of the coefficients of these two predictors revealed that they were not statistically significant (p > 0.05). We then tested an aggregated model (Model 4) including both the N400 and the SRI. This model displayed a λ of 0.59, which was considerably higher compared to the model including the SRI or the N400 only. The inclusion of both the N400 and the SRI yielded a considerable increase in the predictive ability of the model, with a λ in the multivariate model almost equal to the sum of the λs of the two univariate models. A classic voting model (Model 5) including socio-demographic variables had a good fit, but was a worse predictor than the aggregated model with the N400 and the SRI. Finally, the pooled model (Model 6) had a remarkable predictive power and confirmed the robust effect of both the N400 and the SRI.

**Table 1:** Effects of selected predictors on voting for a populist or mainstream party.

	Model 1	Model 2	Model 3	Model 4	Model 5	Model 6
N400 economy	1.084** (0.332)			1.407** (0.470)		2.822* (1.417)
IAT		− 0.0438 (0.253)				− 0.907 (0.610)
SRI economy			2.200*** (0.583)	2.681*** (0.756)		5.168** (1.911)
Age					0.516 (0.354)	− 0.420 (0.801)
Age squared					− 0.00691 (0.00520)	0.00531 (0.0115)
Gender (male)					1.872** (0.721)	3.809* (1.717)
Interest in politics					− 0.836 (0.574)	0.862 (1.730)
Left–right self-placement					0.735*** (0.221)	2.102* (0.862)
Constant	− 0.440 (0.284)	− 0.341 (0.252)	0.354 (0.329)	− 0.352 (0.390)	− 11.73* (5.539)	− 10.60 (15.21)
Observations	65	65	65	65	65	65
McFadden pseudo-*R*^2^	0.156	0.000	0.353	0.494	0.299	0.769
Nagelkerke pseudo-*R*^2^	0.257	0.001	0.512	0.658	0.450	0.872
λ (Adj. correctly predicted count)	0.222	0.000	0.444	0.593	0.407	0.815

Standard errors in parentheses; *p < 0.05, **p < 0.01, ***p < 0.001.

## Discussion

The investigation of political attitudes using measurements of brain activity has gained popularity in recent years^11–13^. fMRI and EEG studies have revealed differences in brain structure and function depending on political preferences^12–19,36^. However, in the absence of a comparison across different methods, it is difficult to determine whether brain data captures those preferences better than more traditional methods of attitude assessments, such as self-reports and implicit behavioral tests. Moreover, as the extant literature has mainly focused on the liberal-conservative political spectrum, it is unclear whether other political divides would be reflected in different patterns of brain activity. Here, we resolved these issues by comparing different implicit and explicit methods of attitude assessment and by focusing our investigation on populist and non-populist attitudes.

We show that features associated with populist or non-populist narratives are processed rapidly in the brain. The political content expressed by the items in terms of the two narratives was decoded from brain activity starting at 400 ms after word onset. In the same latency region, and consistent with our previous investigation conducted within the context of the EU referendum in the UK^19^, we observed a modulation of the N400 in response to survey items that contradicted one’s political attitudes. Specifically, the N400 was larger for survey items expressing populist views in voters of mainstream parties, and larger for non-populist survey items in voters of populist parties. The N400 is generally thought to reflect the difficulty of semantic access^47^. Accordingly, the N400 is reduced for items that are semantically incongruent within a given context^26,48,49^. These congruency effects are not limited to word meanings, but also involve world knowledge gained through experience^50^, including social world knowledge^27–30^. We speculate that political beliefs associated with populist and non-populist narratives are organized in rich knowledge structures in semantic memory. The large N400 response to items that contradicted one’s political views may signal that it is more difficult to access this politically-charged attitude information that is stored in long-term memory^28,49^. Another view on the N400 is that the component reflects the ease with which a word is integrated in the preceding semantic context^51^. In our study, the integration of the last, critical word with the context of the preceding sentence could have been more effortful when the political valence—attributed by the last word—contradicted one’s views. For example, the context of the sentence: “The introduction of citizens’ income will be…” may have led a populist voter to expect and pre-activate features that are associated with his/her set of beliefs (e.g., “beneficial”, “valuable”). Words that rendered the sentence incongruent with those beliefs (e.g., “detrimental”) may have been perceived as unexpected, resulting in intensified semantic analysis, more effortful integration with the preceding sentence context, and consequently larger N400 effects. Either way, such processing was likely affectively charged, as posited by the “hot cognition” framework^23,24^, and as suggested by recent findings showing that the evaluation of politically incongruent information involves brain regions implicated in emotion and affect, such as the amygdala^52^.

Regardless of the specific functional significance of the N400, we show here that populist and mainstream beliefs are extracted from political statements as quickly as 400 ms in the brain. As participants were only asked to report their agreement or disagreement with each item, the processing of each item as populist or non-populist was task-irrelevant. This suggests that access to populist or non-populist attitudes likely occurred unintentionally. Thus, our findings support theoretical work positing that political concepts are rapidly and automatically activated from long-term memory upon the presentation of a politically-charged stimulus^21–25^. It should be noted that at the time of data collection, the political debate in Italy was centered around the contraposition between mainstream and populist parties. Furthermore, the country was the first and only nation in Europe with a government composed entirely of populist parties. Therefore, attitudes associated with populist and mainstream parties were most likely encapsulated in political preferences and key drivers of voting choice in our sample. Altogether, we show that differences in brain responses as a function of political preferences are not limited to long standing political ideologies, but extend to other, relatively new political divides, such as those introduced by populism.

An interesting and unexpected finding of the current investigation is that the differences in brain responses to populist and non-populist content first emerged for economy survey items, and only at around 1100 ms for culture-related items, which covered issues such as immigration and European cultural integration. Of course, it should be noted that this finding requires replication in future studies before any firm conclusions are drawn. However, one hypothesis is that—at least in the electoral context of this study—economic content was more affectively-charged and somehow salient for the brain, thus having processing priority. This hypothesis merges theoretical frameworks from both social neuroscience and political science. According to the iterative-reprocessing model^25^, affectively valenced social stimuli are initially processed in limbic structures, followed by more controlled reprocessing of the stimulus which is supported by cortical structures. Interestingly, EEG evidence suggests that the reprocessing of a stimulus starts at around 800 ms^53^, which is consistent with the later onset of effects for culture items that is observed here. Furthermore, political science theories have emphasized the relevance of economic considerations in voting decisions^54–56^. Italy has been in a prolonged state of economic crisis, characterized by job insecurity and unemployment, increasing inequality and uncertain prospects. This has generally resulted in widespread anger in sections of the population^57^, especially in younger generations. Thus, voters might weigh economic issues more heavily in their evaluations, particularly at times of economic crisis^58^, and possibly in association with a higher affective charge. While admittedly preliminary, our findings strongly suggest that brain responses may be faster for political issues that are particularly salient in a given electoral context. Surprisingly, neural representations of anti-establishment survey items, which are assumed to tap into the core features of populism, did not differ between populist and non-populist items at any latency interval. We found no difference in the SRI for anti-establishment items between voters for populist and mainstream parties, which may suggest that in our sample, that mainly consisted of young adults, this populist sentiment was similar across participants.

The comparison of implicit and explicit predictors of electoral choice revealed an overall advantage of explicit predictors. However, our data showed that brain responses to economic survey items—as indexed by the magnitude of the N400 effect—predicted future voting behavior, especially in combination with the SRI. The SRI and the N400 together accounted for a 59% proportional reduction of prediction error, which can be considered an excellent predictive ability^59^, superior to models of voting behavior tested in similar electoral contexts^60^. The N400 added an incremental predictive power to the explicit measurement of attitudes, suggesting that the combination of an explicit index of agreement with electoral survey items and the measurement of the N400 results in more valid predictions of voting behavior. Contrary to our expectations, IAT scores did not predict future voting choice. This is surprising given that several studies showed that IAT scores could predict voting choices^8–10^*.* One explanation could be that our category stimuli were candidate pictures. Since our dependent variable was party vote, party logos would have been more effective in the context of the current investigation. Although, it should be noted that previous studies showed no difference between candidate-IAT and party-IAT scores when both formats were used within the same experiment^9,10^, so we find it unlikely that the IAT format had an influence on the results. Another explanation could be that the categorization task itself was more cognitively demanding compared to previous studies, resulting in more controlled processing. In previous IAT studies, subjects were asked to categorize faces of political leaders based on their identity^10,61^, whereas in the current investigation, we asked participants to categorize the faces of politicians into leaders of populist or mainstream parties. Moreover, previous IAT studies used candidates that could be mapped onto a well-defined left–right (or Republican-Democrat) political divide. The attribution of party candidates to populist and mainstream parties may have been less straightforward for some participants. Future studies aimed at exploring populism could compare N400 effects with measures of implicit attitudes that bypass an explicit categorization of populist vs non-populist parties, such as the Affect Misattribution Procedure^62^ used in previous voting behavior research^63^.

Altogether, our findings show that the measurement of brain activity can capture politically relevant dimensions beyond the traditional liberal-conservative divide, and can provide valuable insights into the core features of populist and non-populist narratives. Although traditional surveys have pragmatic advantages over the measurement of brain activity (easier to distribute, cost- and time-efficient), here we show that the additional costs of including a neuroscientific measurement are justified by an increase in prediction accuracy. This may have important implications for election polling and campaign evaluations. This line of research is still at its infancy, but the notion that the brain activity of a few individuals can be used to forecast others’ choices has already received empirical support. Studies have demonstrated that brain activity in a laboratory sample could forecast aggregate lending, crowdfunding choices, purchase behaviors, and smoking behavior in response to media ads and news sharing on the internet (reviewed in^64^). Note that our study was not designed to develop a new generalizable brain-based method to predict future election outcomes; we used a small sample, which was not selected to be representative of the Italian (or any other) population. However, our results, together with the findings reviewed above, provide a proof-of principle suggesting that it may be possible in future studies to use neural activity to forecast aggregate voting choice.

## Materials and methods

### Participants

We collected data from 82 participants (45 females; M_age_ = 24 years; SD_age_ = 7) during the 5 weeks preceding the EP election in Italy (May 26, 2019). Participants were professionals or students on different BSc and MSc courses, aged between 18 and 55 years, with normal or corrected-to-normal vision and eligible to vote. All participants gave their written informed consent before starting the experiment and received €20 for their participation. The study was approved by Sapienza University of Rome Ethics Committee and was conducted according to institutional ethical provisions and the Declaration of Helsinki.

### Materials

#### Survey task

**Figure 4:**
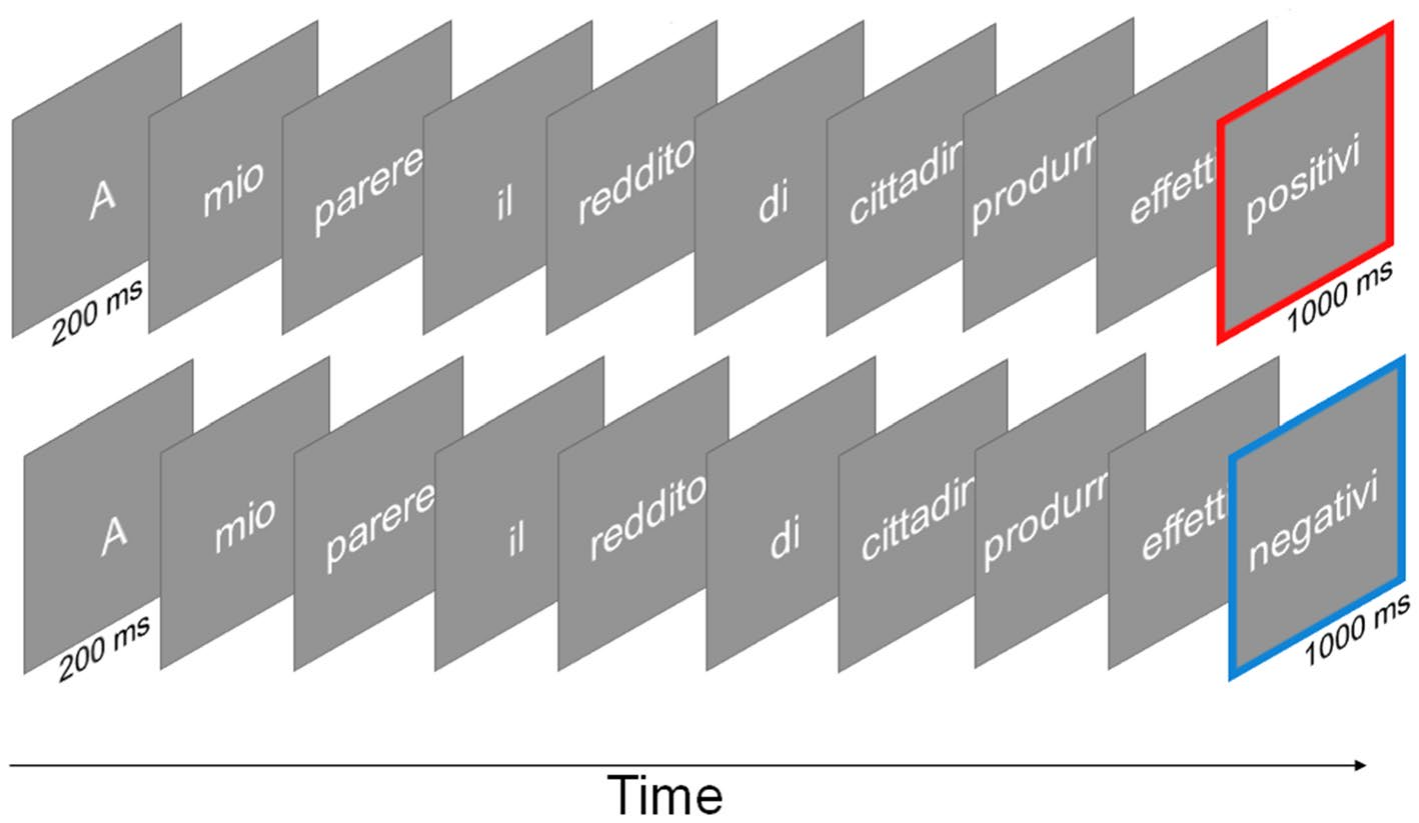
Task. Schematic illustration of a populist (above, *I believe t that citizen’s income will have beneficial effects*) and a non-populist (below, *I believe that citizen’s income will have negative effects*) trial.

Stimuli were 126, 10-word survey items selected from the Eurobarometer survey (https://ec.europa.eu/commfrontoffice/publicopinionmobile/), the European Social Survey (http://www.europeansocialsurvey.org/), the Issue Competition Comparative Project^65^ and other sources. Items were adapted for the current experiment to reflect populist and non-populist views and to achieve the target number of words. There were 42 survey items for each of three issue dimensions: anti-establishment, economy and culture. These are classically considered fundamental dimensions to organize political attitudes and voting choices^66^. For each issue dimension, half of the items were designed to reflect populist views, while the other half were designed to reflect non-populist views. Supplementary Table S3 shows an example of a populist and a non-populist survey item for each issue dimension. In each pair, the two items were identical except for the last word (critical word), which was an antonym, thus attributing the opposite meaning to the statement (Fig. 4). Before the experiment, items were pre-tested on an independent group of 41 participants (all eligible to vote, 20 females, M_age_ = 40 years; SD_age_ = 11) to evaluate whether they reflected a populist or a non-populist view. In this pre-test, participants were given a list of 182 survey items and were asked to judge whether each item supported the view of a populist or a non-populist party. The 126 items that we selected for the actual experiment were the ones that received the highest agreement among participants.

#### IAT

Political category stimuli were head-only faces of the leaders of the four main Italian parties at the time of the research development, specifically two mainstream parties (Silvio Berlusconi/Forza Italia, Nicola Zingaretti/Partito Democratico) and two populist parties (Luigi Di Maio/Movimento Cinque Stelle, Matteo Salvini/Lega). Evaluative stimuli were five positively-valenced and five negatively valenced words. Positively valenced words were gioia (joy), fortuna (luck), felicità (happiness), serenità (serenity), and piacere (pleasure). Negatively-valenced words were orribile (horrible), dolore (pain), pericolo (danger), brutto (awful), and disastro (disaster).

### Procedure

#### Survey task

Upon arrival to the lab, participants were asked to fill out a questionnaire regarding their political preferences, including their interest in politics, whether they leaned towards the political left or the political right and their voting intention. Next, participants were fitted with the EEG cap and sat in front of the screen for the computer-based task. The task was identical to our previous study^19^. A schematic illustration of a trial is shown in Fig. 4. Survey items were presented word-by-word at the center of the screen. Trials began with a 1000 ms fixation mark, and then each word appeared for 200 ms, followed by a 300 ms blank; this is considered a typical stimulus onset asynchrony in N400 studies^67^. The last word (critical word) was presented for 1000 ms, and the inter-item interval varied randomly between 3500 and 4000 ms. Words were presented in white Helvetica on a grey background using the Cogent 2000 toolbox (http://www.vislab.ucl.ac.uk/cogent.php). Participants were asked to indicate whether they agreed with the item by pressing a key on the keyboard, either the far-left key with their left index finger, or the far-right key with their right index finger. The response hand that indicated agreement or disagreement was counterbalanced across participants. The order of items was randomized anew for each participant. Fourteen practice trials preceded the beginning of the experiment.

#### IAT

At the end of the task, participants performed the IAT^7^. Following the procedure used in a previous work^9^, the IAT procedure consisted of seven blocks of trials (see Supplementary Table S4). Participants were required to categorize the faces into leaders of a mainstream or a populist party and the words into positive or negative, separately or in combined blocks. Participants were told to give their response as quickly as possible using the far-right and far-left keys of the keyboard. As shown in Supplementary Table S4, during the first combined blocks (block 3 and 4), one political category and one evaluative category shared the same response key (for instance, Mainstream and Positive), and the two remaining categories shared the other key (Populist and Negative). During the second combined blocks (block 6 and 7), the response hand assignment was reversed for the political category but not for the evaluative category (following on from the previous example, Mainstream and Negative shared the same key). Note that the administration of the task was not based on the voting intention of the participants, therefore the IAT did not include a priori congruent and incongruent blocks. The difference in response times during these blocks is referred to as the IAT effect. The rationale is that if responses are faster, say, for Mainstream and Positive, an implicit preference is inferred for Mainstream. In all blocks, evaluative stimuli were presented in black color on a white background. Category labels remained in the upper corners of the screen for the whole duration of the task and were presented in red (words) or blue (political leaders) color. The order of combined blocks (i.e. Mainstream/Positive and Populist/Negative vs. Mainstream/Negative and Populist/Positive) was counterbalanced across participants.

Participants were contacted again via phone or email in the weeks following election day to report their vote.

### EEG recording and pre-processing

EEG was acquired with a 64-channel system (ANT Neuro, Enschede, the Netherlands). Electrodes were positioned according to an equidistant montage using an elasticated cap (https://www.ant-neuro.com/sites/default/files/images/waveguard_layout_064ch.png). Electrodes CPz and AFz served as online reference and ground, respectively. Two additional electrodes were placed on the left and right mastoids, and one electrode was placed below the left eye to record the electrooculogram (EOG). EEG signals were digitized at 2048 Hz. Electrode impedances were kept below 5 kΩ. Offline analyses were conducted using EEGLAB^68^, ERPLAB^69^ and the Decision Decoding ToolBOX^38^ (DDTBOX; see multivariate pattern analyses below). Data were digitally filtered between 0.1 and 30 Hz and channels with excessive artifacts were interpolated. Data were then re-referenced to the average reference and the EEG continuous recording was segmented into epochs. Epochs were time-locked to the onset of the critical word, and lasted from 100 ms prior to the onset of the critical word to 1200 ms afterwards. A 100 ms pre-stimulus baseline was applied. Ocular and muscular artifacts were corrected with independent component analysis (ICA)^70^, and after ICA correction, epochs were further scrutinized to remove artifacts still contaminating the data. Participants with less than 14 artifact-free epochs in the relevant conditions were excluded from further analyses (one populist voter and one mainstream voter).

### EEG analyses

#### Standard event-related potential analysis

ERP waveforms were computed by averaging artifact-free epochs surrounding the last, critical word as a function of item type (populist vs non-populist survey items) and political issue dimension (anti-establishment, economy or culture). Across the three issue dimensions, the mean number of trials after artifact rejection was 20.2. Mean ERP amplitudes were then extracted in the 300–600 ms time window for the following 12 posterior electrodes: CP1, CP2, CP3, CP4, P1, P2, P3, P4, Pz, PO3, PO4, POz. The choice of time-window and electrodes was based upon both the inspection of the grand-averaged ERPs, and previous reports^19,71^. We conducted an omnibus mixed model Analysis of Variance (ANOVA) with Voting Behavior (two levels: Mainstream, Populist) as between-subjects factor, and the within-subjects factors: Item Type (two levels: Populist, Non-Populist), Issue Dimension (three levels: Anti-Establishment, Economy, Culture) and Electrode (12 levels), with separate follow-up ANOVAs for each issue dimension and voting group. The Greenhouse–Geisser correction was applied if the assumption of sphericity was violated, and in this case the uncorrected degrees of freedom (df), the corrected P values, and the correction factor ε are reported. The α level was set to 0.05.

#### Multivariate pattern analyses

The aim of MVPA was to predict whether each given item expressed populist or non-populist views from patterns of brain activity. We reasoned that political issues that were experienced as highly relevant would be associated with stronger agreement or disagreement—as a function of underlying political beliefs—and would therefore be more strongly represented in neural patterns and, as a consequence, increase the accuracy for predicting the item type from brain activity.

MVPA was conducted on the same epoched and baseline-corrected data used for the standard component analysis. The data was first separated by political issue dimension (anti-establishment, economy, and culture) and then sorted by item type (populist and non-populist items) within each political issue. Because these analyses used a cross-validation procedure (see below), a minimum of 20 trials per issue and item type was required. Two subjects were excluded due to an insufficient number of trials, leading to a final sample size of N = 67. Three independent analyses were conducted for each participant, with one analysis for each political issue dimension. DDTBOX^38^ was used to predict whether each given item was a populist or a non-populist item from spatially distributed patterns of ERPs, which utilized a sliding window approach by moving a 10 ms analysis time window through the trial in non-overlapping steps. Each analysis time window contained 20 time-points × 61 channels (= 1220 features), and the data from all trials that were extracted from these windows constituted the spatio-temporal neural patterns used for this prediction. For each participant, three independent analyses were conducted, one for each political issue dimension. For each analysis, these data patterns were randomly sorted into ten sets, each containing the same number of trials from populist and non-populist items. If one condition had more trials than the other, trials were randomly drawn to match the number of trials in the condition with less trials. This procedure is common practice to circumvent possible drawing biases^38,72^. Note that it remains possible that other attributes of the data, such as systematic differences in variance in relevant channels, biased our classifications. However, these might be relevant discriminatory features of the data, and (as in previous studies) could not be controlled for a priori. A tenfold cross-validation procedure was implemented, and first a linear support vector machine (SVM) classifier (with a fixed regulation parameter C = 1) was trained on the trials associated with each item type from nine of the ten sets using LIBSVM^73^. Based on this training data, the classifier estimated a decision boundary (hyperplane) in feature space that optimally separated patterns associated with the two item types. The trained classifier was then tested on the data from the left-out set, which was used as the test set. This procedure was repeated ten times, with each data set serving as the test data once, whilst independently training the classifier again on the other nine sets. In addition, to circumvent any possible biases that may have occurred due to data sorting, the entire tenfold cross-validation procedure was itself repeated ten times, each time with a new random allocation of trials to the ten sets. The average of all 10 × 10 classification analyses constituted the final prediction accuracy for the respective analysis time window, and indicated how well populist and non-populist items could be classified based on this small analysis time window alone. The analysis time window was then shifted by 10 ms to capture the next brief period of the trial, and the entire procedure was repeated for each analysis time window until the end of the epoch was reached. The final result from each set of analyses (one for each political issue category) was an “information time course”, which reflects how well populist and non-populist items could be predicted across the trial for the respective category.

Given the complexity of statistical testing using an arbitrary number of analysis time steps, using standard tests against a theoretical chance level has been argued to be sub-optimal^74^. Furthermore, the analysis time steps were not truly independent, given that they captured brief periods of sustained ERPs. Thus, we generated a distribution of empirical chance results by repeating all ten iterations of the tenfold cross-validation analysis with the same data and the same labels (i.e. associations with item type) for each participant and each political-issues category in each analysis time window, but the assignment of labels to data was randomly shuffled^38,72^. Group-level statistical testing was performed against this empirical shuffled-labels (i.e. chance) distribution for each separate analysis window, thus providing a conservative estimate of statistical significance. Group-level statistical tests for each political issue dimension (anti-establishment, economy, culture) were conducted across the whole sample. Based on our previous investigation^19^, we expected differences in the N400 temporal region of interest (ROI, 300–600 ms) and conducted analyses in this region using t-tests (α = 0.05). Our further analyses examined whether any other time windows outside the N400 ROI contained predictive information. Consequently, corrections for multiple comparisons were performed using cluster-based permutation tests based on the cluster mass statistic (cluster inclusion alpha = 0.05, permutation samples = 5000). This approach takes advantage of the statistical non-independence of adjacent analysis time windows, but is not as overly conservative as the Bonferroni correction. This analysis revealed the general information time-course of survey item processing for each of the three political issues separately. All data are available on the Open Science Framework website (https://osf.io/8n2zh/).

## Supplementary Information

Supplementary Information.
