# Supplementary material for: Early EEG responses to pre-electoral survey items reflect political attitudes and predict voting behavior
Source: Sci Rep. 2021 Sep 21;11:18692. doi: 10.1038/s41598-021-96193-y (PMC8455561; doi:10.1038/s41598-021-96193-y)
Supplement: Supplementary file 1 — Supplementary Information. [file 41598_2021_96193_MOESM1_ESM.pdf]

Supplementary Material for

Early EEG responses to pre-electoral survey items reflect political attitudes and predict voting behavior

Giulia Galli, Davide Angelucci, Stefan Bode, Chiara De Giorgi, Lorenzo De Sio, Aldo Paparo, Giorgio Di Lorenzo, Viviana Betti

Contents:

- Supplementary Table S1: Task performance-Reaction times
- Supplementary Table S2: Multivariate pattern classification results
- Supplementary Table S3: Example of items
- Supplementary Table S4: Blocks of the Implicit Association Test

Supplementary Table S1: Task performance-Reaction times

	Survey Item	
	Populist	Non-populist
**Issue**	Mean (SD)	Mean (SD)
Anti-establishment	1720 (324)	1711 (304)
Economy	1731 (318)	1745 (290)
Culture	1732 (322)	1677 (312)

Note: N = 67; all reaction times expressed in milliseconds

Supplementary Table S2: Multivariate pattern classification results

	Anti-Establishment		Economy		Culture	
Time-step	*t-value*	*p-value*	*t-value*	*p-value*	*t-value*	*p-value*
-100	0.273	.393	-0.586	.720	-1.097	.862
-90	1.191	.119	0.234	.408	0.020	.492
-80	0.927	.179	-1.041	.849	-0.565	.713
-70	0.898	.186	-2.031	.977	1.335	.093
-60	0.342	.367	-1.227	.888	0.692	.246
-50	0.551	.292	0.493	.312	-1.655	.949
-40	0.319	.375	-1.448	.924	0.053	.479
-30	0.607	.273	-0.903	.815	-1.026	.846
-20	0.269	.395	0.073	.471	0.425	.336
-10	0.560	.289	-0.249	.598	1.387	.085
0	1.567	.061	0.878	.192	-0.347	.635
10	0.270	.394	0.476	.318	-1.304	.902
20	1.346	.091	0.281	.390	-1.153	.873
30	-0.188	.574	0.613	.271	-1.556	.938
40	-0.826	.794	0.966	.169	-0.623	.732
50	1.941	**.028**	-0.782	.781	-0.643	.739
60	1.117	.134	0.403	.344	-0.706	.759
70	1.691	**.048**	0.542	.295	0.293	.385
80	0.634	.264	-0.032	.513	0.379	.353
90	0.658	.256	-0.018	.507	0.343	.366
100	0.955	.172	-0.019	.507	1.224	.113
110	-0.087	.535	-0.255	.600	0.705	.242
120	-0.517	.697	0.675	.251	0.942	.175
130	-1.074	.857	0.057	.477	0.473	.319
140	0.183	.428	0.473	.319	0.988	.163
150	-0.719	.763	-0.333	.630	-0.500	.691
160	-0.236	.593	0.599	.276	0.364	.358
170	-1.054	.852	-1.038	.849	0.348	.365
180	0.403	.344	-0.628	.734	-0.236	.593
190	1.899	**.031**	-2.082	.979	1.352	.091
200	0.918	.181	-0.821	.793	1.157	.126

210	-0.426	.664	-0.344	.634	0.167	.434
220	-0.406	.657	0.131	.448	1.229	.112
230	-0.295	.615	-0.142	.556	0.466	.321
240	-1.229	.888	0.578	.282	-0.026	.510
250	-1.629	.946	0.744	.230	-0.400	.655
260	-0.452	.674	-0.154	.561	-1.072	.856
270	1.420	.080	-0.203	.580	-0.566	.713
280	-0.421	.663	-0.472	.681	0.170	.433
290	-0.025	.510	-1.358	.910	1.145	.128
300	0.598	.276	-2.011	.976	1.619	.055
310	0.204	.419	-1.837	.965	0.933	.177
320	-0.175	.569	-0.559	.711	-0.211	.583
330	0.513	.305	-1.039	.849	-0.372	.644
340	-0.113	.545	0.701	.243	-1.561	.938
350	0.600	.275	0.378	.353	-1.543	.936
360	-0.391	.651	1.080	.142	-0.146	.558
370	-0.790	.784	0.818	.208	1.407	.082
380	-0.293	.615	-0.021	.508	-0.605	.726
390	-1.054	.852	1.558	.062	-0.664	.746
400	-0.373	.645	2.104	**.020**	1.303	.098
410	-1.369	.912	0.470	.320	-0.594	.723
420	-0.006	.503	0.901	.186	1.179	.121
430	0.046	.482	2.807	**.003**	-0.157	.562
440	0.589	.279	2.392	**.010**	-1.559	.938
450	0.289	.387	2.431	**.009**	-0.736	.768
460	0.189	.425	2.485	**.008**	-0.156	.562
470	-0.214	.584	1.818	**.037**	0.068	.473
480	-0.117	.546	-0.251	.599	1.011	.158
490	-0.285	.612	0.334	.370	-0.781	.781
500	-1.274	.896	-0.502	.691	-0.033	.513
510	-1.530	.935	0.270	.394	-0.259	.602
520	-0.702	.757	1.830	**.036**	0.460	.324
530	-0.326	.627	1.275	.103	1.098	.138
540	-1.220	.887	1.411	.081	0.131	.448

550	-1.840	.965	1.053	.148	-0.279	.609
560	-1.189	.881	-0.087	.535	1.230	.112
570	-1.377	.913	0.699	.244	0.320	.375
580	-1.520	.933	0.431	.334	0.082	.467
590	-1.603	.943	0.653	.258	1.478	.072
600	-1.667	.950	0.914	.182	1.583	.059
610	-2.326	.988	0.912	.183	3.112	**.001**
620	-0.338	.632	1.296	.100	2.940	**.002**
630	-0.359	.640	0.941	.175	0.399	.346
640	-1.156	.874	-0.655	.743	0.454	.326
650	-1.073	.856	0.568	.286	0.559	.289
660	-1.880	.968	0.746	.229	1.562	.062
670	0.163	.436	1.618	.055	-0.047	.519
680	-1.036	.848	2.202	**.016**	0.145	.443
690	-0.673	.748	2.188	**.016**	0.312	.378
700	-2.280	.987	2.291	**.013**	0.007	.497
710	-2.150	.982	0.555	.290	1.543	.064
720	-1.182	.879	1.083	.141	1.247	.108
730	-0.882	.809	1.299	.099	1.836	**.035**
740	-0.941	.825	1.510	.068	0.701	.243
750	-0.665	.746	0.652	.258	2.419	**.009**
760	-0.842	.798	-0.376	.646	2.507	**.007**
770	-1.482	.928	-0.844	.799	2.584	**.006**
780	-0.973	.833	0.406	.343	2.129	**.018**
790	-0.777	.780	1.713	**.046**	-0.229	.590
800	-0.386	.650	1.076	.143	0.728	.235
810	-0.969	.832	1.319	.096	-0.062	.525
820	-0.659	.744	-0.509	.694	1.286	.101
830	-1.310	.903	-2.646	.995	2.581	**.006**
840	0.584	.281	-1.199	.883	1.412	.081
850	-0.042	.517	-0.358	.639	1.643	.053
860	0.567	.286	0.693	.245	0.934	.177
870	-0.331	.629	0.309	.379	-0.256	.601
880	-0.606	.727	0.163	.435	-0.417	.661

890	-1.070	.856	0.301	.382	1.827	**.036**
900	0.410	.342	0.119	.453	0.748	.229
910	-0.670	.748	0.588	.279	1.440	.077
920	0.542	.295	-0.129	.551	2.353	**.011**
930	-0.148	.559	0.379	.353	1.830	**.036**
940	-0.681	.751	-0.022	.509	-0.476	.682
950	-1.588	.941	0.513	.305	-0.114	.545
960	-1.690	.952	-0.854	.802	0.037	.485
970	-0.974	.833	-0.577	.717	-0.173	.569
980	-0.735	.768	0.749	.228	0.710	.240
990	-0.167	.566	1.427	.079	1.652	.052
1000	-0.912	.818	-0.369	.643	0.822	.207
1010	-0.573	.716	0.713	.239	1.257	.107
1020	-0.497	.690	1.297	.100	0.442	.330
1030	-1.333	.907	2.047	**.022**	1.801	.038
1040	-1.533	.935	1.164	.124	3.197	**.001**
1050	0.050	.480	0.237	.407	1.989	**.025**
1060	0.985	.164	0.191	.424	0.852	.199
1070	1.627	.054	0.288	.387	0.802	.213
1080	-0.619	.731	1.318	.096	2.474	**.008**
1090	-1.762	.959	0.838	.203	1.996	**.025**
1100	0.251	.401	0.154	.439	2.230	**.015**
1110	0.512	.305	0.727	.235	2.140	**.018**
1120	0.299	.383	0.826	.206	2.931	**.002**
1130	-0.773	.779	1.661	.051	3.814	**<.001**
1140	-1.165	.876	2.766	**.004**	2.453	**.008**
1150	-0.628	.734	0.658	.256	2.562	**.006**
1160	-0.829	.795	-0.030	.512	2.443	**.009**
1170	-0.658	.744	0.734	.233	2.346	**.011**
1180	-0.181	.572	2.370	**.010**	3.902	**<.001**
1190	-0.565	.713	1.194	.118	2.833	**.003**
1200	-0.862	.804	1.343	.092	2.170	**.017**
1210	0.299	.383	0.368	.357	2.140	**.018**
1220	-0.525	.699	-0.941	.825	2.974	**.002**

Note: N = 67; analysis time windows contained data from 10 ms (20 data points) and 61 channels to predict mainstream vs. populist survey items for three issue dimensions separately (anti-establishment; economy; culture); significant (uncorrected) time windows are in bold; uncorrected clusters (minimum two time windows) are highlighted in light grey; clusters that additionally survived cluster-correction are highlighted in dark grey.

Supplementary Table S3: Examples of survey items.

**Survey Item**	**Issue dimension**	
Populist	Anti-establishment	Per il nostro futuro le elite rappresentano sicuramente un problema<br>*(Elitism is a problem for the future of our country)*
Populist	Economy	A mio pare il reddito di cittadinanza produrrà effetti positivi<br>*(I believe that citizen's income will have beneficial effects)*
Populist	Culture	La libera circolazione delle persone nell'UE è un fatto negativo<br>*(Freedom of movement within the EU is undesirable)*
Non-populist	Anti-establishment	Preferisco essere rappresentato da un politico che da un cittadino<br>*(I would rather be represented by a citizen than a politician)*
Non-populist	Economy	Fare sacrifici per ridurre il debito pubblico è auspicabile<br>*(Making sacrifices to reduce public debt is beneficial)*
Non-populist	Culture	Chiudere i porti alle navi con i profughi è inaccettabile<br>*(Closing ports to migrant rescue vessels is unacceptable)*

Supplementary Table S4: Blocks of the Implicit Association Test (Greenwald et al., 1998).

Block	Type of judgement	Left Key	Right Key	Number of trials
1	Political leaders' categorization	Mainstream	Populist	20
2	Word categorization	Positive	Negative	20
3	Combined categorization	Mainstream/positive	Populist/negative	18
4	Combined categorization	Mainstream/positive	Populist/negative	40
5	Political leaders' categorization	Negative	Positive	20
6	Combined categorization	Populist/positive	Mainstream/negative	18
7	Combined categorization	Populist/positive	Mainstream/negative	40
